# Sex-Specific Dietary Predictors of Blood Glucose Identified Through Decision Tree Modeling in Adults

**DOI:** 10.3390/nu17193119

**Published:** 2025-09-30

**Authors:** Joanna Gautney, Christina Aguilar, Julian Chan, David Aguilar

**Affiliations:** 1 Department of Sociology and Anthropology, Weber State University, 1299 Edvalson St., Ogden, UT 84403, USA; joannagautney@weber.edu; 2 Health, Physical Education, and Recreation Department, Weber State University, 1435 Village Dr., Ogden, UT 84403, USA; christinaaguilar@weber.edu; 3 Department of Mathematics, Weber State University, 1415 Edvalson St., Ogden, UT 84403, USA; julianchan@weber.edu; 4 Department of Exercise and Nutrition Sciences, Weber State University, 1435 Village Dr., Ogden, UT 84403, USA

**Keywords:** blood glucose, dietary fiber, energy expenditure, dietary fat, linoleic acid, alpha-linolenic acid, machine learning

## Abstract

Background/Objectives: Diabetes mellitus is a global public health crisis, with cases projected to rise to 1.3 billion by 2050. Lifestyle interventions are crucial in preventing and managing Type 2 diabetes. This study used a machine learning approach to explore the relationship between dietary components and fasting blood glucose in a young adult population, with a focus on potential sex-specific differences. Methods: This cross-sectional study analyzed data from 288 young adults (195 females, 93 males; mean age 23 years). Participants provided two-day diet records, and their fasting capillary blood glucose was measured. Machine learning was used to predict blood glucose based on a variety of dietary variables, including fiber, macronutrient proportions, and fat types. Energy expenditure was used as a proxy for energy intake. Models were created for the overall population, males, and females. Results: In the overall population, the most important predictor of fasting blood glucose was fiber intake. For females, the most important predictor was energy expenditure, followed by fat quality (linoleic to alpha-linolenic acid ratio and saturated fat intake). For males, the most predictive factor was the percentage of calories from fat, followed by alpha-linolenic acid intake. Conclusions: The findings suggest that predictors of blood glucose differ between males and females, highlighting the need for sex-specific strategies in blood glucose management. The models emphasize the importance of increasing fiber intake, maintaining a healthy energy intake, and improving fat quality by prioritizing essential fatty acids. This approach can be used to inform personalized dietary recommendations for the prevention and management of diabetes.

## 1. Introduction

Diabetes mellitus is a common chronic illness with which over 800 million adults worldwide live [1]. Individuals with diabetes tend to have higher all-cause mortality and are at increased risk for debilitating complications, including renal failure, vision loss, and amputation [1,2]. Diabetes is the seventh leading cause of death in the United States and is associated with other chronic illnesses, such as cardiovascular disease and some cancers, and is also associated with mortality due to acute diseases such as respiratory illness [2]. Diabetes already places a significant burden on healthcare systems, and new estimates project that the number of people living with diabetes will rise to at least 1.3 billion worldwide by 2050 [3].

Type 2 diabetes, which accounts for 90–95% of all cases, typically develops in adulthood and is largely preventable through lifestyle intervention when caught early enough [3]. It is caused by elevated blood glucose levels and metabolic dysregulation, leading to insulin resistance [2]. Impaired fasting glucose has long been identified as an early risk factor for the development of diabetes, even in the absence of other risk factors, and doctors can diagnose the disease with a fasting plasma glucose test. Even within the conventionally defined normal range, higher fasting plasma glucose levels are independently linked to a greater risk of developing diabetes, indicating that the likelihood of onset rises progressively with increasing glucose levels, regardless of other risk factors [4,5].

Researchers have identified a strong genetic component to the development of prediabetes and diabetes. Hundreds of genes have been studied for their association with impaired glucose regulation, and while the results have shown high variability in the association of those genes with the development of the disease, a positive family history of type 2 diabetes has been shown to nearly double the individual risk of developing the disease [6,7]. Lifestyle interventions can lower risk, particularly in younger individuals [8,9,10,11]. Physical and dietary modifications have been shown to have strong potential to improve blood glucose management [12].

In recent years, researchers have increasingly turned to machine learning to model diabetes risk based on various health, diet, and activity parameters [13,14,15,16,17]. Previous research has also demonstrated the effectiveness of decision tree modeling for predicting metabolic outcomes. Saltzgiver et al. showed that sex-specific dietary and activity factors could predict metabolic syndrome components in young adults using classification trees [18]. Other investigators have applied similar approaches to larger cohorts, finding decision tree methods effective in identifying predictors of incident metabolic syndrome and diabetes risk [19,20]. These findings reinforce the value of tree-based models in uncovering nonlinear associations and generating interpretable rules in metabolic disease research. Therefore, we sought to use a novel approach of predictive modeling to assess factors associated with glucose levels. We used decision tree regression, a type of machine learning model, to examine the relationships between several diet and activity variables and their ability to predict blood glucose values. These variables include activity levels, macronutrient proportions in the diet, as well as types of fats, essential fatty acids, protein, and dietary fiber consumption. Dietary fat is of special interest as fatty acids influence glucose metabolism by affecting plasma membrane and enzyme function, gene expression, and insulin signaling [21]. Essential fatty acids, including alpha-linolenic and alpha-linoleic acids, have been shown to have a modulating effect on blood glucose and improve insulin sensitivity [12,21,22,23]. We hypothesized that diet and activity variables, particularly fiber, protein, essential fatty acids, and energy expenditure, would be predictive of blood glucose levels, and that predictive factors may differ by gender.

Three models were generated to assess variables associated with blood glucose levels: an overall population model, a female population model, and a male population model. For the overall population, dietary fiber consumption, energy expenditure, linoleic acid consumption, and fat percentage of total calorie intake predict blood glucose values. Our model also showed that the variables most strongly predicting blood glucose differ when divided by gender. For females in the study population, energy expenditure was the most important variable for predicting blood glucose, followed by essential fatty acid ratio and saturated fat consumption. For males in the study population, the percentage of calories from fat in the diet, followed by consumption of alpha-linolenic acid, was the strongest predictor.

Predicting early risk factors for the development of diabetes, such as elevated blood glucose, can be a useful tool in the prevention and management of the disease. Models such as these may be useful in designing diabetes diets geared towards prevention or management of the disease, even in relatively young and healthy populations, and may be even more impactful in older populations already living with diabetes.

## 2. Materials and Methods

### 2.1. Data Analysis and Collection Methods

This cross-sectional study (IRB# 16-ED-062) analyzed data from Weber State University students enrolled in an introductory nutrition course. A total of 288 participants (93 males, 195 females) aged 18–65 years (mean age: 23 years) volunteered for the study.

Participants provided two-day dietary records and underwent anthropometric measurements, including height, weight, body mass index (BMI), and waist circumference (WC).

Capillary blood glucose was measured after a minimum 10 h overnight fast using fingerstick sampling and the Alere Cholestech LDX Analyzer (Alere Inc., Waltham, MA, USA). This device employs a glucose oxidase enzymatic method, provides results within ~5 min, and has a reportable range of 20–500 mg/dL. Intra- and inter-assay CVs are <5%, and accuracy was ensured through daily calibration and manufacturer-provided quality control standards.

### 2.2. Diet Records

Participants recorded their usual food and water intake over two consecutive days using Diet and Wellness Plus Software (Cengage, Boston, MA, USA). Dietary variables analyzed included total energy intake, estimated energy expenditure, intake of grains, vegetables, fruits, dairy, and protein foods, as well as macronutrient composition (% of calories from carbohydrates, protein, and fat), fat types (saturated, monounsaturated, polyunsaturated, trans, and unspecified fats), and essential fatty acids (linoleic acid [18:2] and linolenic acid [18:3]).

Calculated total daily energy expenditure (TDEE) was used as a proxy for energy intake since self-reported dietary energy intake is known to be unreliable, with systematic underreporting of up to 30–40% [24,25]. In contrast, energy intake estimated from calculated TDEE aligns more closely with doubly labeled water measures and provides a more valid marker of true intake [26,27,28].

### 2.3. Statistical Analysis

Analyses were performed with the Python programming language version 3.11.13 using statistical and graphics packages. The libraries Scikit-learn version: 1.6.1, Numpy version: 2.0.2, and Pandas version: 2.2.2 were used for the analysis of the data.

### 2.4. Regression Trees

Machine Learning regression trees were created to predict blood glucose. Missing data for any of the variables resulted in the observation being dropped from the data set. The regression trees are designed to prevent statistical overfitting and to produce generalizable results via pruning by controlling the depth of the tree, the minimum number of data points in each branch or node, and the number of nodes. In addition, this statistical practice has the added benefit of providing more practical recommendations to individuals. The standard metric for error the model uses is the RMSE (root mean square error). In the context of linear regression, this metric can be used to estimate the standard deviation of the model and can be useful in understanding the predictions of the model. We also calculated the R-squared statistic, which represents the amount of variation the model explains of the data, and can also be used for model evaluation [29,30,31].

The tree-building process follows a “top-down, greedy” approach known as recursive binary splitting. At each successive split, the algorithm selects the variable and corresponding cutoff value that yields the greatest possible reduction in error. This selection is an automated procedure inherent to the machine learning algorithm and is not predetermined by the researchers. We input the variables of food group consumption and energy expenditure for the model to obtain these predictions. This analysis provides a visual and hierarchical representation of the most important inputs for prediction. In this way, the regression trees provide different ways to decrease glucose. Each cut point is associated with the greatest reduction in error and clinical implications based on the sample data. Regression trees have several advantages: when there is a nonlinear or complex relationship between the input variables and the response variable, regression trees generally outperform less flexible models such as linear regression for the given input variables. Regression trees closely mirror human decision-making processes and are easy to explain. While our sample size is modest, the model nonetheless provides valuable insights and illustrates multiple dietary pathways that can influence glucose regulation.

## 3. Results

### 3.1. Participant Characteristics

A total of 288 young adults (195 females, 93 males; mean age 23 years) were included. Descriptive intake data show the variability in food component intake (Table 1).

### 3.2. Overall Model, Decision Tree with Whole Population Included

The data set used to produce this tree has 200 observations obtained from the full data set. We selected the variables related to foods, fats, and lifestyle. We dropped rows of data with missing observations and dedicated 75% of the data to building this regression tree and 25% of the data for model evaluation. The regression tree, being a top-down greedy algorithm, indicates that fiber is the most important variable, and low fiber intake increases the risk of high glucose levels. Under the scenario of higher fiber intake, the next most important variable is energy expenditure, with higher energy intake associated with higher blood glucose levels and lower energy expenditure associated with lower blood glucose levels. Next, if energy expenditure is high, then the percentage of total calories from fat becomes significantly predictive, with a higher percentage of calories from fat resulting in higher fasting glucose. Alternatively, if energy expenditure is lower, then linoleic acid percentage of calories becomes significantly predictive, with higher linoleic acid consumption associated with lower fasting glucose. We calculated the RMSE as 11.58 (mg/dL glucose). Additionally, we calculated the R-squared statistic to be 18.41%, which corresponds to a 42.9% correlation. The R-squared statistic was significant at the 1% level of significance.

### 3.3. Sex-Stratified Model: Male

Based on the whole population model, it was determined that fat and energy were the most important nutrients to focus on, outside of fiber. The consumption table suggested that there were different eating patterns for males and females (Table 1). Therefore, we computed models based on gender. We separated the data set for subsequent analysis, exclusively for males, and after removing missing values, 83 observations remained. Due to the smaller sample size, we used the entire data set for the building and evaluation of the regression tree and found that the RMSE for this regression tree was 7.4 (mg/dL glucose). The R-squared statistic was 15.76%, corresponding to a 39.69% correlation. The R-squared statistic was significant at the 1% level of significance.

First, the percentage of calories from fat is the most important predictor, with a higher percentage of calories from fat resulting in lower fasting glucose. Under higher fat consumption, the percentage of calories from linoleic acid is predictive, with a higher percentage of calories from linoleic acid resulting in higher blood glucose. Under lower fat consumption, alpha-linolenic acid mitigates the risk of higher glucose levels, with lower levels predicting higher blood glucose.

### 3.4. Sex-Stratified Model: Female

When the data set was divided by gender, the analysis for females had 182 observations. We used the entire data set to generate and evaluate the tree, as we did for the male regression tree, to estimate the error in the model. We calculated the RMSE as 9.37 (mg/dL glucose), and the R-squared statistic was 16.83%, corresponding to a 41.02% correlation. The R-squared statistic was significant at the 1% level of significance.

For females, the most important factor is energy expenditure, with higher energy expenditure resulting in higher blood glucose. The next most important factor is fat consumption. In those with lower energy expenditure, the linoleic to alpha-linolenic (LA/ALA) ratio is predictive, with a lower ratio resulting in lower blood glucose. Alternatively, for those with higher energy expenditure, the percentage of calories from fat is predictive, with higher calories from fat resulting in higher blood glucose. In those with lower calories from fat, lower monounsaturated fat results in higher blood glucose.

## 4. Discussion

Dietary variables analyzed included total energy intake, estimated energy expenditure, intake of grains, vegetables, fruits, dairy, and protein foods, macronutrient composition (% of calories from carbohydrates, protein, and fat), fat types (saturated, monounsaturated, polyunsaturated, trans, and unspecified fats), and essential fatty acids (linoleic acid [18:2] and linolenic acid [18:3]). Among these, the most predictive factors were fiber intake, fat intake, and fat type. Low fiber intake consistently emerged as a primary contributor to elevated glucose levels, while under sufficient fiber intake, other variables shaped outcomes: under lower energy expenditure, higher linoleic acid intake predicted lower blood glucose, whereas under higher energy expenditure, lower total fat intake predicted lower blood glucose. Because males and females differ in energy metabolism, lipid oxidation, and essential fatty acid requirements [32,33,34], fat intake and fat type were examined separately for each gender. This approach provided clearer insight into how different dietary fats, including essential fatty acids, affect glucose regulation across sex-specific metabolic contexts [35], with the three trees together illustrating the complex interactions among fiber, energy balance, and fat quality in shaping glucose outcomes. Two-day diet records do not fully capture habitual intake and are subject to day-to-day variability and reporting error; however, such error likely biases results toward the null, making our observed associations conservative. Given our focus on population-level prediction rather than exact long-term intake, the standardized protocol and decision-tree approach remain appropriate for identifying dietary predictors of glucose [36,37].

### 4.1. Overall Population Model

**Figure 1:**
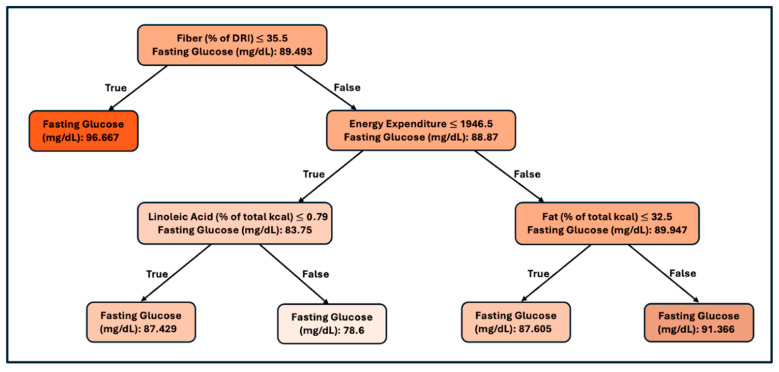
Regression tree predicting fasting blood glucose in the overall study population. Regression tree predicting fasting blood glucose (mg/dL) in the overall study population (*n* = 200 after listwise deletion). Each node represents a decision split based on the predictor variable and its cutoff value, with the corresponding mean fasting glucose (mg/dL) for that branch. Terminal nodes (boxes at the bottom) indicate predicted mean fasting glucose for participants in that subgroup. Variables selected by the algorithm included dietary fiber intake (as % of DRI), energy expenditure (kcal/day), percentage of total energy from fat, and percentage of total energy from linoleic acid. DRI, Dietary Reference Intake.

In the total population model, the first split in the tree was determined by the fiber percentage of the daily reference intake (DRI). Consumption of less dietary fiber (<35.5% of DRI) was associated with elevated glucose (Figure 1). Dietary fiber consumption has been shown to be associated with improved glycemic control. Fiber that is both readily fermentable and forms viscous gels slows digestion by changing how macronutrients interact with digestive enzymes and slowing glucose absorption, promoting satiety and lowering the glycemic index of meals [38,39,40,41,42].

Among those consuming higher levels of dietary fiber, the next important factor was energy expenditure. In this analysis, energy expenditure was used as a proxy for energy intake, and energy intake has been positively correlated with blood glucose level and insulin resistance [43,44]. In individuals with lower energy expenditure (≤1946.5 kcal per day), a higher intake of linoleic acid (≤0.79% of total kcal) had a protective effect against elevated glucose. Consumption of linoleic acid has been shown to improve insulin sensitivity and modulate blood glucose, and while the exact mechanism is not known, it is hypothesized that linoleic acid has anti-inflammatory effects that protect against elevated blood glucose [21,45,46].

Among those with higher energy expenditure, the analysis showed that total fat intake was predictive of blood glucose level. Those consuming higher fat levels (total kcal from fat < 32.5%) had a higher risk of higher blood glucose values. Excess dietary fat increases circulating triglycerides and free fatty acids, impairing insulin signaling and glucose uptake in muscle and liver. This reduces glucose clearance from the blood, promoting hyperglycemia and insulin resistance [47] (Figure 1).

### 4.2. Male Fasting Glucose Decision Tree

**Figure 2:**
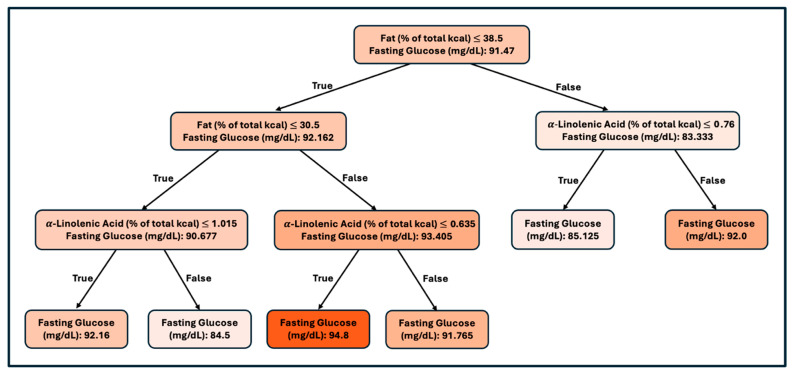
Regression tree predicting fasting blood glucose in males. Regression tree predicting fasting blood glucose (mg/dL) in males (*n* = 83). The first split was the percentage of energy from fat, followed by α-linolenic acid (ALA, % of energy). Terminal nodes display predicted mean fasting glucose for each subgroup.

In males, the first separation was determined by the proportion of energy from fat, indicating that the percentage of fat from total calories was the most important factor influencing glucose outcomes (Figure 2). Meta-analyses have shown that low-fat diets do not consistently lead to reductions in fat mass, and high-fat diets can sometimes result in favorable body composition when paired with carbohydrate restriction [48]. Thus, the decision tree’s branching structure reflects this complexity, as fat intake may exert both protective and adverse effects depending on accompanying nutrient composition. Among those with lower or adequate fat intake (≤~30.5% of energy), the next differentiating factor was the percentage of calories from alpha-linolenic acid. A higher intake of alpha-linolenic acid was associated with more favorable glucose levels, consistent with evidence that alpha-linolenic acid enhances insulin sensitivity, improves membrane fluidity, and reduces inflammation [49,50]. This pattern is particularly noteworthy as it mirrors the Acceptable Macronutrient Distribution Range (AMDR), which recommends keeping total fat intake within 20–35% of total energy while emphasizing the inclusion of essential fatty acids for optimal health.

In contrast, among males with higher fat intake (>~38.5% of energy), alpha-linolenic intake no longer appeared to influence outcomes. Instead, this outcome reflects the metabolic adaptations associated with very high-fat, low-carbohydrate diets such as ketogenic diets, which increase fat oxidation, suppress insulin, and enhance satiety despite high fat consumption. Taken together, the most favorable glucose outcomes were observed with moderate fat consumption combined with higher alpha-linolenic acid intake (Figure 2). This finding reinforces current dietary guidelines that emphasize moderation in total fat consumption while ensuring adequate intake of essential fatty acids [51].

### 4.3. Female Fasting Glucose Decision Tree

**Figure 3:**
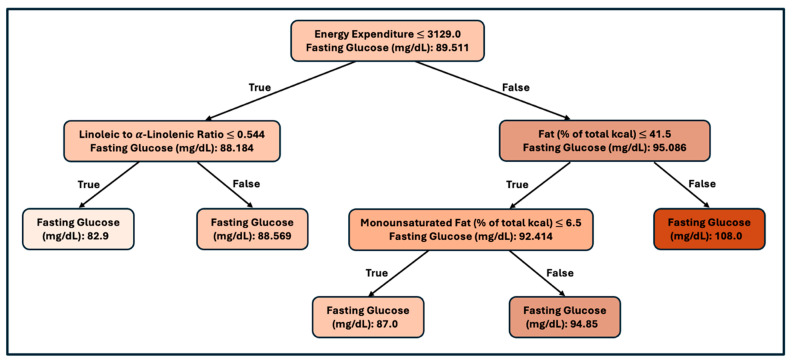
Regression tree predicting fasting blood glucose in females. Regression tree predicting fasting blood glucose (mg/dL) in females (*n* = 182). The first split was energy expenditure, followed by the linoleic to α-linolenic acid (LA:ALA) ratio, percentage of energy from total fat, and percentage of energy from monounsaturated fat (MUFA). Terminal nodes indicate predicted mean fasting glucose for each subgroup.

In females, the initial separation was driven by energy expenditure, which served as a proxy for energy intake. This suggests that overall energy intake was the most predictive factor influencing glucose outcomes (Figure 3). Among those with lower expenditure (≤~3129 kcal), the next key factor was the linoleic to alpha-linolenic acid ratio. A low LA/ALA ratio (≤0.544) was associated with lower glucose levels, whereas a higher ratio (>0.544) predicted higher glucose levels (Figure 3). This aligns with evidence that alpha-linolenic acid exerts anti-inflammatory effects by competing with linoleic fatty acid for enzymatic conversion into eicosanoids. A higher omega-3 intake shifts the balance toward less pro-inflammatory lipid mediators, thereby reducing systemic inflammation, improving insulin receptor signaling, and enhancing membrane fluidity [49,50]. Conversely, a low omega-3–omega-6 ratio favors arachidonic acid-derived pro-inflammatory eicosanoids, which are linked to insulin resistance and impaired glucose uptake.

Among those with higher expenditure (>~3129 kcal), the omega-3–omega-6 ratio was no longer predictive, suggesting that greater energy intake may reduce or overshadow its influence. Similarly to the first tree with the entire population, higher fat intake (>~41.5% of calories) was associated with higher fasting glucose levels. Within the lower fat group, monounsaturated fat intake further distinguished outcomes; The link between monounsaturated fat intake above 6.5% of energy and less favorable glucose outcomes should be viewed with caution. Higher monounsaturated fat intake may indicate nutrient displacement, where increases in monounsaturated fats may reduce intake of omega-3 fatty acids or complex carbohydrates, offsetting expected benefits. A threshold effect is also possible; monounsaturated fats improve cardiometabolic health when replacing saturated fat or refined carbohydrate [50,52], but under conditions of high total fat intake, additional monounsaturated fats may contribute mainly to excess caloric load without added benefit.

Taken together, the findings suggest that under lower caloric intake, the balance between omega-3 and omega-6 fatty acids is most relevant for glucose regulation, whereas under higher energy intake, the quality and amount of dietary fat, particularly monounsaturated fats, becomes the dominant factor.

## 5. Conclusions

This study demonstrates that predictors of blood glucose differ between males and females, emphasizing the need for sex-specific strategies in diabetes prevention and management. Males show a higher prevalence of type 2 diabetes at younger ages and lower body fat mass, whereas females exhibit a greater risk factor burden later in life [53]. These findings highlight the importance of increasing dietary fiber intake, decreasing energy intake, and improving fat quality by replacing saturated with unsaturated fats to increase intake of essential fatty acids, while ensuring total fat intake does not exceed the Acceptable Macronutrient Distribution Range (AMDR) for percentage of calories from fat. Replacing saturated fats with unsaturated fats has been shown to lower blood glucose and reduce diabetes risk [21]. Accordingly, foods rich in vegetable oils, seeds, and nuts should be prioritized, while limiting animal-derived saturated fats. The model further suggests that integrating demographic, lifestyle, and biomarker data can support personalized dietary recommendations for both diabetes prevention and management.

## Figures and Tables

**Table 1:** Nutrient and dietary intake measures of study participants.

Nutrient/Measure	Female (Mean ± SD)	Male (Mean ± SD)	Overall (Mean ± SD)
Fasting Glucose (mg/dL)	89.46 ± 10.16	91.25 ± 8.17	90.02 ± 9.60
Energy (kcal)	2436.41 ± 870.47	3244.86 ± 1104.77	2698.38 ± 1023.53
Grains (servings)	5.02 ± 2.55	6.85 ± 3.16	5.63 ± 2.89
Vegetables (servings)	1.22 ± 1.27	0.92 ± 0.92	1.12 ± 1.17
Dairy products (servings)	1.39 ± 1.25	1.99 ± 1.55	1.58 ± 1.38
Protein foods (servings)	4.97 ± 3.97	6.69 ± 5.32	5.53 ± 4.52
Fruits (servings)	0.99 ± 1.12	0.90 ± 1.13	0.96 ± 1.12
Carbohydrates (% of total kcal)	50.85 ± 9.53	50.12 ± 9.79	50.61 ± 9.60
Protein (% of total kcal)	17.09 ± 5.19	16.58 ± 5.49	16.92 ± 5.28
Fat (% of total kcal)	33.75 ± 7.01	34.55 ± 8.25	34.01 ± 7.43
Sugar (kcal)	386.87 ± 209.07	463.05 ± 288.96	411.47 ± 239.97
Fiber (% of DRI)	71.84 ± 43.14	67.09 ± 45.97	70.30 ± 44.03
Saturated fat (g)	21.88 ± 9.59	29.84 ± 14.54	24.45 ± 11.99
Monounsaturated fat (g)	18.74 ± 10.01	25.90 ± 12.23	21.05 ± 11.27
Polyunsaturated fat (g)	11.45 ± 6.58	16.60 ± 11.95	13.11 ± 8.99
Trans fat (g)	0.61 ± 0.99	0.76 ± 0.97	0.66 ± 0.99
Unspecified fat (% of total kcal)	7.55 ± 4.20	6.84 ± 4.00	7.32 ± 4.14
Monounsaturated fat (% kcal)	9.49 ± 3.97	9.49 ± 3.25	9.49 ± 3.74
Polyunsaturated fat (% kcal)	5.88 ± 2.89	6.08 ± 2.68	5.94 ± 2.82
Saturated fat (% kcal)	11.02 ± 3.71	10.66 ± 3.07	10.90 ± 3.51
Trans fat (% kcal)	0.22 ± 0.51	0.18 ± 0.42	0.21 ± 0.48
Linoleic acid (% kcal)	0.81 ± 0.55	0.81 ± 0.81	0.81 ± 0.65
Alpha-linolenic acid (% kcal)	0.84 ± 0.97	0.83 ± 0.95	0.84 ± 0.96
Sugar (g)	96.72 ± 52.27	115.76 ± 72.24	102.87 ± 59.99

Values are presented as mean ± standard deviation (SD). Intakes are expressed as absolute amounts (grams, kilocalories, or servings), percentages of total daily energy intake, or percentages of the Dietary Reference Intake (DRI), as specified.

## Data Availability

The original contributions presented in this study are included in the article/Supplementary Material. Further inquiries can be directed to the corresponding author.

## Supplementary Materials

The following are available online at https://www.mdpi.com/article/10.3390/nu17193119/s1, raw participant data: MasterData.csv, Demographics_final.py, final_female_tree.py, final male_fat.py, foods_final_tree.py.
